# Transition Education for Young Adults With Type 1 Diabetes: Pilot Feasibility Study for a Group Telehealth Intervention

**DOI:** 10.2196/10909

**Published:** 2018-11-05

**Authors:** Anastasia Albanese-O'Neill, Giovanna Beauchamp, Nicole Thomas, Sarah C Westen, Nicole Johnson, Desmond Schatz, Michael J Haller

**Affiliations:** 1 Division of Pediatric Endocrinology Department of Pediatrics University of Florida Gainesville, FL United States; 2 Division of Pediatric Endocrinology Department of Pediatrics University of Alabama at Birmingham Birmingham, AL United States; 3 Department of Clinical and Health Psychology College of Public Health and Health Professions University of Florida Gainesville, FL United States; 4 JDRF New York, NY United States

**Keywords:** diabetes education, mobile phone, telehealth, type 1 diabetes, young adult, transition

## Abstract

**Background:**

Young adults with type 1 diabetes (T1D) experience a decline in glycemic outcomes and gaps in clinical care. A diabetes education and support program designed for young adults was delivered through group videoconference and mobile Web.

**Objective:**

The objective of our study was to assess the feasibility, acceptability, and preliminary efficacy of the program as measured by attendance and webpage views, satisfaction, and pre- and postintervention psychosocial outcomes, respectively.

**Methods:**

Young adults aged 18-25 years were recruited to attend five 30-minute group diabetes education videoconferences during an 8-week period. Videoconferences included an expert presentation followed by a moderated group discussion. Within 48 hours of each videoconference, participants were sent a link to more information on the study website. Feasibility was assessed using data on videoconference attendance and webpage views. Acceptability was assessed via a Satisfaction Survey completed at the conclusion of the study. Descriptive statistics were generated. Preliminary efficacy was assessed via a survey to measure changes in diabetes-specific self-efficacy and diabetes distress. Pre- and postintervention data were compared using paired samples *t* tests.

**Results:**

In this study, 20 young adults (mean age 19.2 [SD 1.1] years) attended an average of 5.1 (SD 1.0) videoconferences equivalent to 153 (SD 30.6) minutes of diabetes education per participant during an 8-week period. Average participant satisfaction scores were 62.2 (SD 2.6) out of a possible 65 points. A total of 102 links sent via text message (short message service) or email resulted in 504 webpage views. There was no statistically significant difference between pre- and postintervention diabetes-specific self-efficacy or diabetes-related distress.

**Conclusions:**

Delivery of diabetes education via group videoconference using mobile Web follow-up is feasible and acceptable to young adults with T1D. This model of care delivery has the potential to improve attendance, social support, and patient-reported satisfaction. Nevertheless, further research is required to establish the effect on long-term psychosocial and glycemic outcomes.

## Introduction

Clinic-based transition support for young adults with type 1 diabetes (T1D) remains inadequate despite expert consensus guidelines published by the American Diabetes Association in 2011 [1-4]. Many young adults with T1D experience gaps in care, have poor glycemic control, and are, thus, at risk for acute and long-term diabetes-related complications. In a large clinical registry based in the United States, only 13% of young adults with T1D achieved the recommended glycated hemoglobin (HbA_1c_) target of 7%, with HbA_1c_ peaking at an average of 9.2% at the age of 19 years [5,6]. This decline in glycemic control occurs as many young adults move away from home and lose access to their established diabetes support system [7,8]. Recent efforts to provide clinic-based structured transition support for young adults with T1D have been associated with improved glycemic control, reduced hypoglycemia, and improved psychological well-being [9,10]. Telemedicine visits have been successfully used in lieu of in-clinic visits to improve adherence to clinical attendance standards for pediatric patients with diabetes in rural settings [11], and an innovative pilot study found group medical appointments for young adults with T1D conducted using Web-based videoconference technology to be feasible and acceptable [12]. However, the use of Web-based group videoconferences to provide a transition-focused diabetes self-management education and support (DSMES) program has not been studied. The purpose of this pilot study was to test the feasibility, acceptability, and preliminary efficacy of a telehealth transition education program designed for young adults with T1D as a first step in our efforts to evaluate its potential for integration into the clinical care paradigm.

## Methods

Following the institutional review board (IRB) approval, participants were recruited via fliers at the University of Florida outpatient diabetes clinic. Upon enrollment, participants completed surveys on paper to obtain demographic data, diabetes history, technology acquisition, and communication preferences. Participants then completed the Problem Areas in Diabetes (PAID) Scale to assess baseline levels of diabetes-specific distress [13,14] and the Confidence in Diabetes Scale (CIDS) to assess baseline levels of diabetes-specific self-efficacy [15]. The PAID Scale consists of 20 items; potential scores range from 0 to 80, with higher scores indicating higher levels of distress. The instrument has demonstrated high internal reliability (Cronbach alpha=.90) as well as reasonable (Spearman ρ=.83) 2-month test-retest reliability and correlates strongly with a wide range of theoretically related psychosocial constructs in diabetes (eg, distress, depression, self-care behaviors, coping, and health beliefs). The CIDS survey has 20 items; potential scores range from 20 to 100, with higher scores indicating higher levels of self-efficacy. The instrument has demonstrated high internal consistency (alpha=.90) and test-retest reliability (ρ=.85, *P*<.001).

Once the surveys were completed, participants were asked to indicate which 5 group diabetes education videoconferences they preferred to attend over the 8-week study period. Each topic was offered a total of 5 times (5 different dates or times). A maximum of 5 participants per videoconference was set to facilitate dialogue and minimize the risk for technical challenges. After participants indicated topical preferences, a study coordinator contacted them to assist with scheduling. Vidyo software (Vidyo, Inc, Hackensack, NJ, USA) was used as the videoconference platform; it allows end users to participate via a smartphone, tablet, laptop, or desktop. Each 30-minute videoconference included a brief (10-15 minute) expert presentation by a pediatric endocrinologist, nurse practitioner (NP), certified diabetes educator (CDE), psychologist, or registered dietitian (RD). At the beginning of each videoconference, a moderator read a scripted, IRB-approved statement regarding privacy and respecting the privacy of all study participants. Only first names were used during group education sessions. Participants had the option to enable or disable video streaming during all videoconferences. The moderator’s introduction was followed by the expert presentation, and then a moderated discussion among participants to foster peer-learning and social support. Table 1 summarizes the diabetes education topics available during the study.

**Figure 1:**
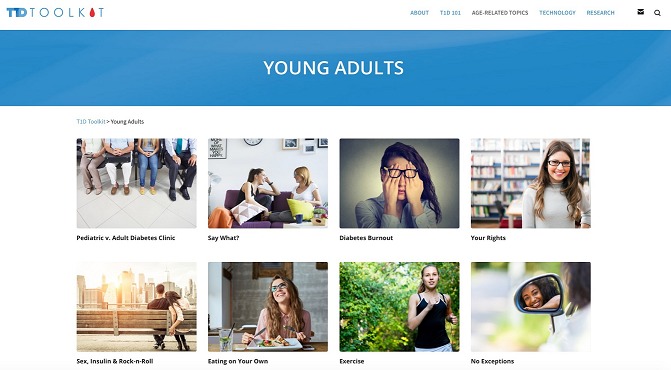
Screenshot of the young adult section of the study website. Source: T1D Toolkit.

Within 48 hours of each videoconference, attendees were sent a link to additional content on a section of the study website specifically for young adults with T1D and their parents (Figure 1). The study website was developed and reviewed by a multidisciplinary team of CDEs, pediatric endocrinologists, NPs, psychologists, RDs, registered nurses, parents, and people with diabetes. The website provides information on basic diabetes management, diabetes technology, and additional content tailored to young adults with T1D. Of note, the website was not publicly available during the study and could only be reached via links sent to participants. Web analytics for the site were monitored and analyzed, with particular attention to relevant page views and increases in website traffic during the 48 hours following the distribution of links via short message service (SMS) text message or email to participants.

At the end of the 8-week study, participants completed the PAID Scale, the CIDS Scale, and a Satisfaction Survey to assess acceptability and usability. The Satisfaction Survey was designed for the study and included 16 questions (13 closed-ended and 3 open-ended) to obtain end-user feedback about the usability and acceptability of the program. Potential scores on the closed-ended questions ranged from 13 to 65, with higher scores indicating higher levels of satisfaction. Answers to open-ended questions were reviewed and coded to inform future iterations of the educational content and clinical model. At the completion of study procedures, a US $75 gift card was provided to participants. Participants were given information about the US $75 gift card at the time of informed consent.

Quantitative data from the Demographic, Communication Preferences, and Satisfaction surveys were analyzed using SPSS, v.25, software (IBM Corp, Armonk, NY) to generate descriptive statistics. Results are expressed as mean (SD) and as frequencies and percentages for categorical variables. Pre- and postintervention scores on the CIDS and PAID scales were analyzed using paired samples *t* test. A *P*<.05 was considered statistically significant. Web analytics were reviewed and pageview data were analyzed to describe participant traffic on the study website, T1DToolkit.org.

**Table 1:** Diabetes education topics.

Videoconference topic	Description
Pediatric versus Adult Diabetes Clinic	What to expect, how to prepare for transition
Say What?	How to have the “Diabetes Talk”
Your Rights	Reasonable work and school accommodations
Sex, Insulin, and Rock-n-Roll	Exploring “taboo” subjects related to real life with type 1 diabetes
Exercise and Nutrition	Optimizing activity and nutrition
Diabetes Burnout and Sources of Support	Identifying burnout, burnout versus depression, and how to find help
New and Emerging Diabetes Technologies	Insulin pumps, continuous glucose monitors, automated insulin delivery

## Results

We approached 21 participants to participate in this study. All enrolled, but 1 participant withdrew immediately following enrollment citing a busy high school sports schedule. Mean age of participants was 19.2 (SD 4.39) years, and 80% (16/21) participants were females. Mean diabetes duration was 10.21 (SD 4.39; range, 2-17) years; mean age at diagnosis was 9 (SD 4.42; range, 3-17) years, and 80% (16/20) participants were insulin pump users. Table 2 provides additional demographic data. All participants had access to a smartphone (20/20, 100%) and most had access to a computer, laptop, or tablet. A majority (9/20, 45%) of participants preferred to receive notifications via both SMS text message and email, with 40% (8/20) preferring SMS text message only and 15% (3/20) email only (Table 3).

Mean attendance was 5.1 (SD 1.0; range, 2-7) diabetes education videoconferences per participant, which is equivalent to an average of 153 (SD 30.6; range, 60-210) minutes of diabetes education per participant. The most popular sessions included Diabetes Burnout (n=17), Your Rights (n=15), Diabetes Technologies (n=15), and Exercise and Nutrition (n=15); these were followed by Transition to Adult Clinic (n=14); Sex, Insulin, and Rock-n-Roll (n=14); and Say What? (n=12). A minimum of 2 and a maximum of 5 participants participated in each of the 35 videoconferences offered during the study. The mean score on the Satisfaction Survey was 62.2 (SD 2.6; range, 57-65). Overall, 95% (19/20) participants responded that they would be “extremely interested” or “very interested” in participating in a similar program in the future. Representative positive responses to the open-ended questions included, “I really enjoyed participating in this study. I got to talk about things that I don’t really talk about with my doctor. And the topics that I have discussed with my doctor, it was interesting to hear different opinions.” Suggestions to improve the program included, “Add a better way to manage all questions from larger groups of people” and “Only one session had technical difficulties where no one could log on. It was fixed quickly.”

The mean scores for diabetes-related distress declined; however, there was no statistically significant reduction when comparing the mean baseline (mean 20.4 [SD 15.0]) and postintervention (mean 17.2 [SD 15.3]) scores on the PAID Scale (*t*_19_=1.04, *P*=.09). Mean scores for diabetes-specific self-efficacy increased; however, there was no statistically significant increase when comparing the mean baseline (mean 87.0 [SD 7.4]) and postintervention (mean 88.2 [SD 6.9]) scores on the CIDS Scale (*t*_19_=−0.79, *P*=.44; Table 4).

**Table 2:** The description of study participants.

Characteristics			Values
Age in years, mean (SD)			19.2 (4.39)
Age at diagnosis in years, mean (SD)			9 (4.42)
Type 1 diabetes duration in years, mean (SD)			10.21 (4.39)
**Insulin regimen, n (%)**			
	Pump	16 (80)	
	Multiple daily injections	4 (20)	
**Gender, n (%)**			
	Male	4 (20)	
	Female	16 (80)	
**Ethnicity, n (%)**			
	Asian	3 (15)	
	Hispanic black	1 (5)	
	Non-Hispanic black	1 (5)	
	Hispanic	1 (5)	
	Non-Hispanic white	14 (70)	
**Residence, n (%)**			
	Independent	14 (70)	
	Parent or guardian	6 (30)	
**Level at school, n (%)**			
	College	17 (85)	
	High School	3 (15)	
**Employment, n (%)**			
	Full time	4 (20)	
	Part time	10 (50)	
	None	6 (30)	

**Table 3:** Technology acquisition and communication preferences.

Characteristics		Values
**Technology acquisition, n (%)**		
	Smartphone	20 (100)
	Tablet	7 (35)
	Laptop	19 (95)
	Desktop	6 (30)
**Communication preference, n (%)**		
	Email only	3 (15)
	Short message service (SMS) text message only	8 (40)
	Email and SMS text message	9 (45)

**Table 4:** Psychosocial outcomes.

Construct	Measure	Mean (SD)			*t* statistic		*df*^a^		*P* value
		Pre	Post						
Diabetes-related distress	Problem areas in Diabetes Scale	20.4 (15.0)	17.2 (15.3)	0.79		19		.09	
Diabetes-related self-efficacy	Confidence in Diabetes Scale	87 (7.4)	88.2 (6.9)	1.04		19		.44	

^a^*df*: degrees of freedom.

**Figure 2:**
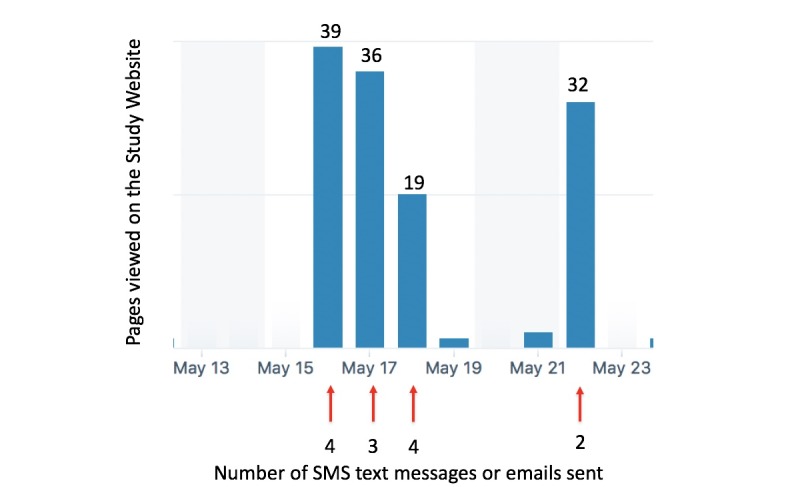
Dose-response example of pushed links and website page views. SMS: short message service.

A total of 102 emails or SMS text messages with links to the study website were sent to participants, resulting in 504 page views. The timing of website page views was strongly linked to the date the links were pushed to participants; this indicates a high level of engagement in the educational content by participants. Figure 2 provides a sample of the dose-response feedback.

## Discussion

This pilot study with young adults with T1D demonstrated high feasibility for providing diabetes education and support via group videoconference and strong participant engagement in Web-based follow-up. Participants reported high levels of acceptability as measured by user satisfaction. In terms of the preliminary efficacy, there was no statistical difference in pre- and postintervention psychosocial outcomes; however, on average, we observed improved scores for both diabetes-related self-efficacy and diabetes-related distress. The findings support results from previous studies that have demonstrated high attendance and satisfaction with individual clinic visits and group medical appointments provided via telehealth to youth and young adults with T1D [11,12].

The implications of these results should be considered in the context of the study’s limitations. Owing to the brief duration of and limited funding for the study, other efficacy-related outcome measures including glycemic control and diabetes knowledge attainment were not assessed. Recruitment took place at a university-based diabetes clinic, where patients may be more highly motivated to attend diabetes education visits. In addition, despite the ubiquity of mobile technology, patients with limited data plans or access to wireless networks may not find participation as feasible without financial support to cover the cost of a mobile data plan.

Nonetheless, the outcomes suggest that this delivery model for diabetes education and support has the potential to increase contact with the clinic, improve access to diabetes education, and provide peer and social support for young adults who have become disconnected from their diabetes network. Future randomized studies that include a control group should explore the intermediate and longitudinal effect of the model on glycemic control, diabetes knowledge attainment, clinic attendance, and psychosocial outcomes. In addition, future studies should measure provider satisfaction and explore the feasibility of reimbursement for telehealth group videoconference education sessions. Convenient, comprehensive, yet tailored diabetes care, education, and support is required to keep young adults engaged in their diabetes management to reduce gaps in care and to mitigate the decline in glycemic control commonly experienced by this patient population. As T1D management becomes more technically complex, videoconference and Web-based models of diabetes care and education delivery can be leveraged to connect patients to providers and educators at a reduced cost with improved convenience and without a decline in patient satisfaction.

## Abbreviations

CDE: certified diabetes educator
CIDS: Confidence in Diabetes Scale
DSMES: diabetes self-management education and support
HbA_1c_: glycated hemoglobin
IRB: Institutional Review Board
NP: nurse practitioner
PAID: Problem Areas in Diabetes Scale
RD: registered dietitian
SMS: short message service
T1D: type 1 diabetes
